# Moxifloxacinium chloride monohydrate

**DOI:** 10.1107/S160053681103707X

**Published:** 2011-09-30

**Authors:** Jing-Jing Qian, Jian-Ming Gu, Jin Shen, Xiu-Rong Hu, Su-Xiang Wu

**Affiliations:** a College of Pharmaceutical Science, Zhejiang Chinese Medical University, Hangzhou, Zhejiang 310053, People’s Republic of China; b Center of Analysis and Measurement, Zhejiang University, Hangzhou, Zhejiang 310028, People’s Republic of China; c Chemistry Department, Zhejiang University, Hangzhou, Zhejiang 310028, People’s Republic of China

## Abstract

The title compound {systematic name: 7-[(1*S*,6*S*)-8-aza-2-azonia­bicyclo­[4.3.0]non-8-yl]-1-cyclo­propyl-6-fluoro-8-meth­oxy-4-oxo-1,4-dihydro­quinoline-3-carb­oxy­lic acid chloride monohydrate}, C_21_H_25_FN_3_O_4_^+^·Cl^−^·H_2_O, crystallizes with two moxi­floxa­cinium cations, two chloride ions and two uncoordinated water mol­ecules in the unit cell. The crystal structure has a pseudo-inversion center except for the chloride ions. In both moxi­floxa­cinium cations, the quinoline rings are approximately planar, the maximum atomic deviations being 0.107 (3) and 0.118 (3) Å. The piperidine rings adopt a chair conformation while the pyrrolidine rings display a half-chair conformation. In the crystal, the carboxyl groups, the protonated piperidyl groups, the uncoordinated water mol­ecule and chloride anions participate in O—H⋯O, O—H⋯Cl and N—H⋯Cl hydrogen bonding; weak inter­molecular C—H⋯O and C—H⋯Cl hydrogen bonding is also present in the crystal structure.

## Related literature

For applications of moxifloxacin hydro­chloride in the medicine field, see: Seidel *et al.* (2000 ▶); Talib *et al.* (2002 ▶); Culley *et al.* (2001 ▶); Liu & Sun (2008 ▶). For the tolerability, solubility, safety and pharmacodynamics of moxifloxacin hydro­chloride, see: Stass *et al.* (1998 ▶); Noel *et al.* (2005 ▶); Varanda *et al.* (2006 ▶). For a related structure of moxifloxacin hydro­chloride methanol solvate, see: Ravikumar & Sridhar (2006 ▶).
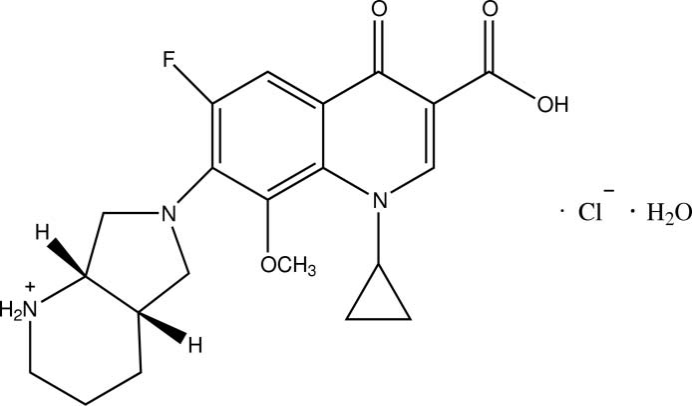


## Experimental

### 

#### Crystal data

C_21_H_25_FN_3_O_4_^+^·Cl^−^·H_2_O
*M* *_r_* = 455.91
Triclinic, 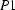

*a* = 6.7280 (3) Å
*b* = 10.6406 (5) Å
*c* = 15.3127 (7) Å
α = 91.7293 (14)°
β = 91.1313 (13)°
γ = 100.8823 (13)°
*V* = 1075.67 (9) Å^3^
*Z* = 2
Mo *K*α radiation
μ = 0.23 mm^−1^
*T* = 296 K
0.49 × 0.37 × 0.22 mm

#### Data collection

Rigaku R-AXIS RAPID/ZJUG diffractometer
Absorption correction: multi-scan (*ABSCOR*; Higashi, 1995 ▶) *T*_min_ = 0.898, *T*_max_ = 0.952
10677 measured reflections
8038 independent reflections
5933 reflections with *I* > 2σ(*I*)
*R*_int_ = 0.024

#### Refinement

*R*[*F* ^2^ > 2σ(*F* ^2^)] = 0.039
*wR*(*F* ^2^) = 0.118
*S* = 1.10
8038 reflections
563 parameters
3 restraints
H-atom parameters constrained
Δρ_max_ = 0.25 e Å^−3^
Δρ_min_ = −0.29 e Å^−3^
Absolute structure: Flack (1983 ▶), 3107 Friedel pairs
Flack parameter: 0.00 (6)

### 

Data collection: *PROCESS-AUTO* (Rigaku, 2006 ▶); cell refinement: *PROCESS-AUTO*; data reduction: *CrystalStructure* (Rigaku, 2007 ▶); program(s) used to solve structure: *SHELXS97* (Sheldrick, 2008 ▶); program(s) used to refine structure: *SHELXL97* (Sheldrick, 2008 ▶); molecular graphics: *ORTEP-3 for Windows* (Farrugia, 1997 ▶); software used to prepare material for publication: *WinGX* (Farrugia, 1999 ▶).

## Supplementary Material

Crystal structure: contains datablock(s) global, I. DOI: 10.1107/S160053681103707X/xu5322sup1.cif

Structure factors: contains datablock(s) I. DOI: 10.1107/S160053681103707X/xu5322Isup2.hkl

Additional supplementary materials:  crystallographic information; 3D view; checkCIF report

## Figures and Tables

**Table 1:** Hydrogen-bond geometry (Å, °)

*D*—H⋯*A*	*D*—H	H⋯*A*	*D*⋯*A*	*D*—H⋯*A*
N1*A*—H12*A*⋯Cl1*B*^i^	0.90	2.26	3.113 (3)	158
N1*A*—H13*A*⋯Cl1*A*	0.90	2.43	3.234 (3)	150
N1*B*—H12*B*⋯Cl1*A*^ii^	0.90	2.22	3.109 (3)	170
N1*B*—H13*B*⋯Cl1*B*	0.90	2.25	3.114 (2)	162
O3*A*—H3*A*⋯O2*A*	0.82	1.74	2.512 (4)	156
O3*B*—H3*B*⋯O2*B*	0.82	1.74	2.508 (4)	155
O5*A*—H51*A*⋯O4*A*	0.82	2.24	3.011 (5)	157
O5*A*—H52*A*⋯Cl1*B*	0.82	2.72	3.422 (5)	144
O5*B*—H51*B*⋯O4*B*	0.82	2.12	2.911 (5)	161
O5*B*—H52*B*⋯Cl1*B*^iii^	0.82	2.41	3.208 (4)	163
C1*A*—H1*A*⋯O3*B*	0.98	2.53	3.373 (4)	144
C2*A*—H21*A*⋯Cl1*A*^iv^	0.97	2.80	3.742 (4)	163
C3*A*—H31*A*⋯Cl1*A*	0.97	2.74	3.518 (5)	138
C3*A*—H32*A*⋯O1*B*^v^	0.97	2.59	3.450 (5)	148
C6*A*—H61*A*⋯O3*B*^vi^	0.97	2.49	3.340 (6)	146
C18*B*—H18*B*⋯O5*A*^vii^	0.98	2.58	3.564 (7)	179

Symmetry codes: (i) 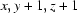; (ii) 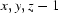; (iii) 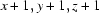; (iv) 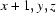; (v) 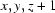; (vi) 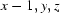; (vii) 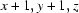.

**Fig. 1.**
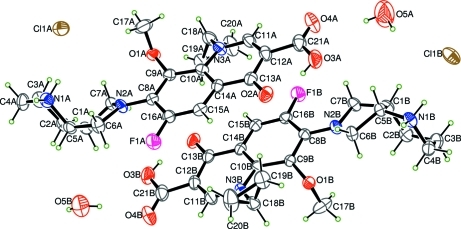
Molecular structure of the title compound (I) showing atom-labelling scheme and displacement ellipsoids at 40% probability level. H atoms are shown as small circles of arbitary radii.

**Fig. 2.**
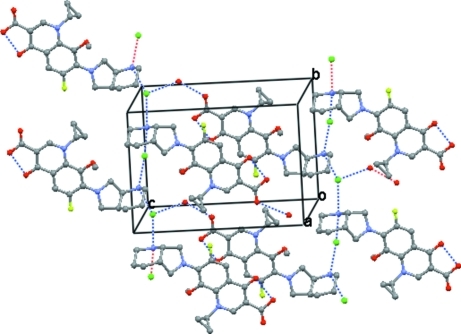
Part of the crystal packing of the title compound. Hydrogen bonds are shown as dashed lines. H atoms not involved in hydrogen bonding have been omitted for clarity.

## supplementary crystallographic information

### Comment

Moxifloxacin hydrochloride, a new fluoroquinolone with a broad spectrum of antibacterial, anaerobes and atypical oranisms (Seidel *et al.*, 2000), is approved by the Food and Drug Administration in December 1999 for use in the treatment of acute bacterial sinusitis, acute bacterial exacerbations of chronic bronchitis, and community-acquired pneumonia caused by susceptible microorganisms (Culley *et al.*, 2001). The crystal structure of moxifloxacin hydrochloride methanol solvate have been reported (Ravikumar & Sridhar, 2006). In the present study, we report the crystal structure of moxifloxacin hydrochloride monohydrate.

The asymmetric unit consists of two independent moxifloxacin cations protonated at the terminal piperidyl N atom, two chloride ions and two lattice water molecules (Fig. 1). The important different of asymmetric unit is the orientation of its piperidinopyrrolidine side chain. In the cation A, the torsion angle of C9—C8—N2—C7 is 35.9 (5)°, while in the cation B, the torsion angle is 168.4 (3)°. So, we can see that the two cations adopt conformations that differ by an almost 180° rotation with respect to the piperidinopyrrolidine side chain. Conformation of the moxifloxacin cations in the structure of title compound and moxifloxacinium chloride-water-methanol solvate (Ravikumar & Sridhar, 2006) shows not much different.

In both moxifloxacin cations of the title compound, the quinoline rings are approximately planar, the maximum atomic deviations being 0.107 (3) and 0.118 (3) Å, respectively. The peridine rings adopt chair conformation with the exposed N atom participating in the hydrogen-bonding interactions, and the pyrrolidine rings favour a half-chair conformation twisted on atoms C1—C5, which is similar to that of moxifloxacinium methanol solvate (Ravikumar & Sridhar, 2006). The cyclopropyl rings are not coplanar with the quinoline ring system, forming the dihedral angles with quinoline ring systems of 73.9 (2) and 74.3 (2)° for cation A and B respectively. The C17—O1 methoxy group is almost perpendicular to the plane of the quinoline ring system [torsion angle of C17—O1—C9—C8 is 94.2 (4)° and -84.1 (4)° for the A and B cations, respectively].

In the crystal structure, different modes of hydrogen-bonding interactions, cation-cation, cation-water, cation-chloride ion and water-chloride ion, stablizing the molecules. Carboxyl atom O3 forms an intramolecular hydrogen bond with carbonyl group O2. This hydrogen bond forms a quasi-six-membered ring. The two H atoms at atom N1 of piperidine ring participate in intramolecular and intermolecular hydrogen bonding with chloride ion. The water molecule acts as a donor in hydrogen bonds with the carbonyl O atom of the carboxylic acid group of cation A, while in the cation B, water molecule forms hydrogen bonds with a chloride ion and the the carbonyl O atom of the carboxylic acid group. In this way, the hydrogen bonds link all of the components of the structure into extented two dimensional networks (Fig.2). Weak intermolecular C—H···O and C—H···Cl hydrogen bonding is also present in the crystal structure.

### Experimental

The crude product is supplied by Zhejiang Jingxin Pharmaceutical Co., LTD. It was recrystallized from ethanol solution, giving yellow crystals suitable for X-ray diffraction.

### Refinement

The difference density indicated the presence of a possible H atom in the atom N1A and N1B, showing that a proton transfer from HCl to amino group of moxifloxacinium molecule. But this H atom was placed in calculated position with N···H = 0.90 Å and refined as riding with *U*_iso_(H) = 1.2*U*_eq_(N). All H atoms of water molecules were located in a difference electron-density map and refined with O—H bond-length restraints of 0.82 (1) Å. Other H atoms were placed in calculated positions with O—H = 0.82 and C—H = 0.93–0.98 Å and included in the refinement in riding model, with *U*_iso_(H)= 1.2*U*_eq_ or 1.5*U*_eq_(carrier atom).

### Crystal data

**Table:**

C_21_H_25_FN_3_O_4_^+^·Cl^−^·H_2_O	*Z* = 2
*M**_r_* = 455.91	*F*(000) = 480
Triclinic, *P*1	*D*_x_ = 1.408 Mg m^−^^3^
Hall symbol: P 1	Mo *K*α radiation, λ = 0.71073 Å
*a* = 6.7280 (3) Å	Cell parameters from 8287 reflections
*b* = 10.6406 (5) Å	θ = 3.1–27.4°
*c* = 15.3127 (7) Å	µ = 0.23 mm^−^^1^
α = 91.7293 (14)°	*T* = 296 K
β = 91.1313 (13)°	Platelet, yellow
γ = 100.8823 (13)°	0.49 × 0.37 × 0.22 mm
*V* = 1075.67 (9) Å^3^	

### Data collection

**Table:**

Rigaku R-AXIS RAPID/ZJUG diffractometer	8038 independent reflections
Radiation source: rolling anode	5933 reflections with *I* > 2σ(*I*)
graphite	*R*_int_ = 0.024
Detector resolution: 10.00 pixels mm^-1^	θ_max_ = 27.5°, θ_min_ = 3.1°
ω scans	*h* = −8→7
Absorption correction: multi-scan (*ABSCOR*; Higashi, 1995)	*k* = −13→13
*T*_min_ = 0.898, *T*_max_ = 0.952	*l* = −19→19
10677 measured reflections	

### Refinement

**Table:**

Refinement on *F*^2^	Secondary atom site location: difference Fourier map
Least-squares matrix: full	Hydrogen site location: inferred from neighbouring sites
*R*[*F*^2^ > 2σ(*F*^2^)] = 0.039	H-atom parameters constrained
*wR*(*F*^2^) = 0.118	*w* = 1/[σ^2^(*F*_o_^2^) + (0.0483*P*)^2^ + 0.3081*P*] where *P* = (*F*_o_^2^ + 2*F*_c_^2^)/3
*S* = 1.10	(Δ/σ)_max_ = 0.002
8038 reflections	Δρ_max_ = 0.25 e Å^−^^3^
563 parameters	Δρ_min_ = −0.29 e Å^−^^3^
3 restraints	Absolute structure: Flack (1983), 3107 Friedel pairs
Primary atom site location: structure-invariant direct methods	Flack parameter: 0.00 (6)

### Special details

**Table:**

Geometry. All esds (except the esd in the dihedral angle between two l.s. planes) are estimated using the full covariance matrix. The cell esds are taken into account individually in the estimation of esds in distances, angles and torsion angles; correlations between esds in cell parameters are only used when they are defined by crystal symmetry. An approximate (isotropic) treatment of cell esds is used for estimating esds involving l.s. planes.
Refinement. Refinement of *F*^2^ against ALL reflections. The weighted *R*-factor *wR* and goodness of fit *S* are based on *F*^2^, conventional *R*-factors *R* are based on *F*, with *F* set to zero for negative *F*^2^. The threshold expression of *F*^2^ > σ(*F*^2^) is used only for calculating *R*-factors(gt) *etc*. and is not relevant to the choice of reflections for refinement. *R*-factors based on *F*^2^ are statistically about twice as large as those based on *F*, and *R*- factors based on ALL data will be even larger.

### Fractional atomic coordinates and isotropic or equivalent isotropic displacement parameters (Å^2^)

**Table:**

	*x*	*y*	*z*	*U*_iso_*/*U*_eq_	
Cl1A	0.08670 (14)	0.50137 (8)	0.99176 (7)	0.0548 (2)	
Cl1B	0.10870 (14)	0.02176 (8)	−0.03926 (7)	0.0567 (3)	
N1A	0.3204 (5)	0.7860 (3)	0.94810 (19)	0.0441 (7)	
H12A	0.2306	0.8388	0.9455	0.053*	
H13A	0.2528	0.7055	0.9375	0.053*	
N1B	0.3516 (4)	0.2937 (2)	0.01252 (17)	0.0400 (6)	
H12B	0.2868	0.3602	0.0099	0.048*	
H13B	0.2680	0.2245	−0.0112	0.048*	
N2A	0.3990 (4)	0.6616 (2)	0.76809 (17)	0.0372 (6)	
N2B	0.5973 (4)	0.3338 (2)	0.22997 (17)	0.0374 (6)	
N3A	0.2638 (5)	0.2658 (2)	0.58859 (19)	0.0416 (7)	
N3B	0.7482 (4)	0.7350 (2)	0.40000 (17)	0.0385 (6)	
O1A	0.2857 (4)	0.3959 (2)	0.75552 (14)	0.0415 (6)	
O1B	0.7298 (4)	0.59852 (19)	0.23736 (14)	0.0383 (5)	
O2A	0.1857 (4)	0.4667 (2)	0.37071 (15)	0.0489 (6)	
O2B	0.8173 (4)	0.5445 (2)	0.62383 (14)	0.0515 (7)	
O3A	0.0877 (5)	0.2578 (3)	0.28571 (17)	0.0638 (8)	
H3A	0.1155	0.3342	0.2996	0.096*	
O3B	0.9164 (5)	0.7573 (3)	0.70351 (17)	0.0669 (8)	
H3B	0.8928	0.6803	0.6913	0.100*	
O4A	0.0747 (5)	0.0744 (3)	0.3508 (2)	0.0770 (9)	
O4B	0.9382 (6)	0.9377 (3)	0.6324 (2)	0.0767 (9)	
O5A	0.0249 (13)	−0.0589 (4)	0.1733 (3)	0.204 (4)	
H51A	0.0738	−0.0201	0.2181	0.306*	
H52A	0.0571	−0.0067	0.1354	0.306*	
O5B	0.8200 (6)	1.0469 (3)	0.7955 (2)	0.0864 (10)	
H51B	0.8621	1.0037	0.7573	0.130*	
H52B	0.8963	1.0577	0.8386	0.130*	
F1A	0.3699 (4)	0.79303 (17)	0.61130 (13)	0.0542 (6)	
F1B	0.6310 (4)	0.20723 (17)	0.38902 (13)	0.0536 (6)	
C1A	0.6328 (5)	0.7360 (3)	0.8836 (2)	0.0393 (7)	
H1A	0.7466	0.7748	0.8482	0.047*	
C1B	0.5301 (5)	0.1742 (3)	0.1187 (2)	0.0359 (7)	
H1B	0.4562	0.0868	0.1061	0.043*	
C2A	0.7169 (6)	0.7224 (3)	0.9757 (2)	0.0483 (9)	
H21A	0.7871	0.6507	0.9753	0.058*	
H22A	0.8149	0.7991	0.9920	0.058*	
C2B	0.7169 (6)	0.2031 (3)	0.0622 (2)	0.0460 (8)	
H21B	0.8035	0.2817	0.0839	0.055*	
H22B	0.7927	0.1344	0.0666	0.055*	
C3A	0.5553 (7)	0.7016 (4)	1.0439 (3)	0.0532 (9)	
H31A	0.4770	0.6154	1.0366	0.064*	
H32A	0.6193	0.7101	1.1017	0.064*	
C3B	0.6593 (6)	0.2168 (4)	−0.0324 (2)	0.0501 (9)	
H31B	0.5806	0.1362	−0.0554	0.060*	
H32B	0.7810	0.2368	−0.0662	0.060*	
C4A	0.4157 (7)	0.7962 (4)	1.0371 (2)	0.0519 (9)	
H41A	0.3116	0.7788	1.0804	0.062*	
H42A	0.4915	0.8823	1.0485	0.062*	
C4B	0.5366 (6)	0.3217 (4)	−0.0419 (2)	0.0473 (9)	
H41B	0.6183	0.4035	−0.0231	0.057*	
H42B	0.4969	0.3268	−0.1027	0.057*	
C5A	0.4756 (5)	0.8203 (3)	0.8792 (2)	0.0398 (8)	
H5A	0.5414	0.9104	0.8872	0.048*	
C5B	0.3902 (5)	0.2698 (3)	0.1065 (2)	0.0356 (7)	
H5B	0.2603	0.2356	0.1328	0.043*	
C6A	0.3868 (7)	0.7958 (3)	0.7874 (2)	0.0500 (9)	
H61A	0.2475	0.8080	0.7849	0.060*	
H62A	0.4650	0.8521	0.7467	0.060*	
C6B	0.4919 (5)	0.3884 (3)	0.1599 (2)	0.0371 (7)	
H61B	0.5864	0.4452	0.1252	0.045*	
H62B	0.3927	0.4348	0.1833	0.045*	
C7A	0.5250 (5)	0.6133 (3)	0.8348 (2)	0.0354 (7)	
H7A1	0.6215	0.5681	0.8078	0.042*	
H7A2	0.4419	0.5566	0.8738	0.042*	
C7B	0.5792 (6)	0.1942 (3)	0.2154 (2)	0.0402 (8)	
H7B1	0.4716	0.1474	0.2496	0.048*	
H7B2	0.7051	0.1673	0.2304	0.048*	
C8A	0.3480 (5)	0.5983 (3)	0.6879 (2)	0.0336 (7)	
C8B	0.6511 (5)	0.3980 (3)	0.3089 (2)	0.0321 (7)	
C9A	0.3091 (5)	0.4642 (3)	0.6802 (2)	0.0340 (7)	
C9B	0.7005 (5)	0.5329 (3)	0.31349 (19)	0.0321 (7)	
C10A	0.2781 (5)	0.3988 (3)	0.5982 (2)	0.0359 (7)	
C10B	0.7331 (5)	0.6013 (3)	0.3943 (2)	0.0330 (7)	
C11A	0.2025 (6)	0.2064 (3)	0.5112 (2)	0.0458 (8)	
H11A	0.1843	0.1175	0.5078	0.055*	
C11B	0.8098 (6)	0.7976 (3)	0.4764 (2)	0.0449 (8)	
H11B	0.8309	0.8866	0.4776	0.054*	
C12A	0.1653 (5)	0.2675 (3)	0.4375 (2)	0.0437 (8)	
C12B	0.8430 (5)	0.7403 (3)	0.5515 (2)	0.0431 (8)	
C13A	0.2009 (5)	0.4047 (3)	0.4385 (2)	0.0393 (8)	
C13B	0.8052 (5)	0.6032 (3)	0.5539 (2)	0.0393 (8)	
C14A	0.2551 (5)	0.4681 (3)	0.5228 (2)	0.0341 (7)	
C14B	0.7501 (5)	0.5352 (3)	0.4709 (2)	0.0337 (7)	
C15A	0.2818 (5)	0.6017 (3)	0.5308 (2)	0.0371 (7)	
H15A	0.2636	0.6488	0.4821	0.045*	
C15B	0.7215 (5)	0.4016 (3)	0.4659 (2)	0.0365 (7)	
H15B	0.7400	0.3567	0.5156	0.044*	
C16A	0.3342 (5)	0.6629 (3)	0.6093 (2)	0.0364 (7)	
C16B	0.6664 (5)	0.3369 (3)	0.3883 (2)	0.0359 (7)	
C17A	0.0780 (7)	0.3606 (5)	0.7779 (3)	0.0736 (14)	
H17D	0.0250	0.4364	0.7915	0.110*	
H17E	0.0673	0.3075	0.8278	0.110*	
H17F	0.0022	0.3143	0.7295	0.110*	
C17B	0.9320 (7)	0.6076 (5)	0.2066 (3)	0.0665 (12)	
H17A	1.0256	0.6601	0.2469	0.100*	
H17B	0.9414	0.6452	0.1502	0.100*	
H17C	0.9643	0.5236	0.2019	0.100*	
C18A	0.3418 (7)	0.1899 (3)	0.6553 (2)	0.0511 (9)	
H18A	0.2446	0.1483	0.6973	0.061*	
C18B	0.6706 (7)	0.8076 (3)	0.3312 (2)	0.0499 (9)	
H18B	0.7667	0.8434	0.2871	0.060*	
C19A	0.5531 (7)	0.2305 (4)	0.6868 (3)	0.0559 (10)	
H19C	0.5842	0.2153	0.7473	0.067*	
H19D	0.6303	0.3086	0.6643	0.067*	
C19B	0.4564 (7)	0.7664 (4)	0.3015 (3)	0.0553 (10)	
H19A	0.3774	0.6921	0.3280	0.066*	
H19B	0.4234	0.7761	0.2405	0.066*	
C20A	0.5007 (8)	0.1171 (4)	0.6256 (3)	0.0723 (14)	
H20A	0.4987	0.0331	0.6489	0.087*	
H20B	0.5449	0.1265	0.5658	0.087*	
C20B	0.5185 (9)	0.8854 (4)	0.3576 (3)	0.0750 (15)	
H20C	0.5232	0.9671	0.3304	0.090*	
H20D	0.4772	0.8831	0.4180	0.090*	
C21A	0.1039 (6)	0.1893 (4)	0.3556 (3)	0.0544 (10)	
C21B	0.9039 (6)	0.8217 (4)	0.6315 (3)	0.0552 (10)	

### Atomic displacement parameters (Å^2^)

**Table:**

	*U*^11^	*U*^22^	*U*^33^	*U*^12^	*U*^13^	*U*^23^
Cl1A	0.0484 (5)	0.0424 (4)	0.0767 (6)	0.0148 (4)	0.0123 (5)	0.0061 (4)
Cl1B	0.0521 (6)	0.0390 (4)	0.0793 (7)	0.0128 (4)	−0.0135 (5)	−0.0130 (4)
N1A	0.0464 (18)	0.0370 (14)	0.0521 (17)	0.0164 (13)	0.0028 (14)	−0.0012 (13)
N1B	0.0443 (17)	0.0328 (13)	0.0438 (16)	0.0115 (12)	−0.0112 (13)	−0.0035 (12)
N2A	0.0485 (17)	0.0249 (12)	0.0387 (15)	0.0097 (12)	−0.0060 (12)	−0.0024 (11)
N2B	0.0508 (18)	0.0281 (12)	0.0340 (14)	0.0102 (12)	−0.0080 (13)	0.0004 (11)
N3A	0.0476 (18)	0.0316 (13)	0.0437 (16)	0.0038 (12)	−0.0070 (13)	−0.0011 (12)
N3B	0.0453 (17)	0.0340 (14)	0.0330 (15)	0.0002 (12)	−0.0019 (12)	−0.0038 (12)
O1A	0.0486 (15)	0.0380 (12)	0.0337 (12)	−0.0028 (10)	−0.0037 (10)	0.0086 (10)
O1B	0.0463 (14)	0.0358 (11)	0.0311 (11)	0.0038 (10)	−0.0016 (10)	0.0033 (9)
O2A	0.0511 (16)	0.0618 (16)	0.0348 (13)	0.0128 (12)	−0.0015 (11)	0.0050 (12)
O2B	0.0528 (17)	0.0706 (17)	0.0293 (13)	0.0073 (14)	−0.0006 (11)	0.0033 (12)
O3A	0.0571 (19)	0.086 (2)	0.0439 (15)	0.0077 (17)	−0.0081 (13)	−0.0173 (15)
O3B	0.073 (2)	0.086 (2)	0.0369 (15)	0.0040 (18)	−0.0013 (13)	−0.0106 (14)
O4A	0.094 (3)	0.0627 (19)	0.069 (2)	0.0093 (17)	−0.0204 (17)	−0.0276 (16)
O4B	0.093 (3)	0.0661 (19)	0.0637 (19)	0.0034 (17)	−0.0099 (17)	−0.0280 (15)
O5A	0.392 (11)	0.087 (3)	0.100 (3)	−0.040 (4)	0.049 (5)	−0.013 (3)
O5B	0.109 (3)	0.081 (2)	0.074 (2)	0.030 (2)	−0.0072 (19)	−0.0076 (17)
F1A	0.0842 (18)	0.0330 (10)	0.0460 (12)	0.0118 (10)	0.0021 (11)	0.0064 (9)
F1B	0.0809 (17)	0.0334 (10)	0.0472 (12)	0.0122 (10)	−0.0024 (11)	0.0074 (9)
C1A	0.0401 (19)	0.0315 (15)	0.0458 (19)	0.0058 (14)	−0.0014 (15)	−0.0023 (14)
C1B	0.0398 (19)	0.0236 (13)	0.0440 (18)	0.0065 (13)	−0.0047 (15)	−0.0031 (13)
C2A	0.047 (2)	0.0431 (18)	0.054 (2)	0.0095 (16)	−0.0125 (17)	−0.0069 (17)
C2B	0.044 (2)	0.0427 (18)	0.054 (2)	0.0164 (16)	0.0001 (17)	−0.0066 (16)
C3A	0.062 (3)	0.051 (2)	0.049 (2)	0.0182 (19)	−0.0073 (19)	0.0047 (17)
C3B	0.055 (2)	0.049 (2)	0.048 (2)	0.0141 (18)	0.0084 (18)	−0.0069 (17)
C4A	0.074 (3)	0.053 (2)	0.0314 (17)	0.0197 (19)	−0.0018 (17)	−0.0018 (15)
C4B	0.061 (2)	0.0436 (18)	0.0364 (18)	0.0079 (17)	0.0037 (17)	−0.0017 (15)
C5A	0.051 (2)	0.0275 (14)	0.0397 (18)	0.0053 (14)	−0.0001 (16)	0.0024 (13)
C5B	0.0384 (19)	0.0332 (15)	0.0355 (17)	0.0086 (13)	−0.0007 (14)	−0.0034 (13)
C6A	0.072 (3)	0.0367 (17)	0.044 (2)	0.0211 (18)	−0.0107 (18)	−0.0006 (15)
C6B	0.052 (2)	0.0306 (15)	0.0308 (16)	0.0152 (15)	−0.0077 (15)	−0.0015 (13)
C7A	0.0419 (19)	0.0315 (15)	0.0334 (17)	0.0093 (14)	−0.0043 (14)	0.0008 (13)
C7B	0.047 (2)	0.0283 (14)	0.0463 (19)	0.0105 (14)	−0.0052 (15)	−0.0022 (14)
C8A	0.0331 (17)	0.0338 (15)	0.0341 (17)	0.0060 (13)	0.0028 (14)	0.0037 (13)
C8B	0.0291 (17)	0.0317 (15)	0.0354 (17)	0.0060 (13)	−0.0002 (13)	0.0000 (13)
C9A	0.0346 (18)	0.0341 (15)	0.0333 (17)	0.0059 (13)	−0.0010 (13)	0.0051 (13)
C9B	0.0353 (18)	0.0329 (14)	0.0274 (15)	0.0042 (13)	0.0000 (13)	0.0024 (12)
C10A	0.0303 (18)	0.0340 (15)	0.0418 (18)	0.0024 (13)	−0.0017 (14)	0.0022 (14)
C10B	0.0289 (17)	0.0330 (15)	0.0356 (17)	0.0026 (13)	0.0004 (13)	−0.0034 (13)
C11A	0.041 (2)	0.0447 (18)	0.047 (2)	0.0008 (15)	−0.0072 (16)	−0.0087 (16)
C11B	0.045 (2)	0.0395 (17)	0.047 (2)	0.0039 (15)	−0.0018 (16)	−0.0113 (16)
C12A	0.0372 (19)	0.0518 (19)	0.0419 (19)	0.0102 (16)	−0.0069 (15)	−0.0109 (16)
C12B	0.0377 (19)	0.054 (2)	0.0360 (18)	0.0064 (16)	0.0007 (15)	−0.0099 (16)
C13A	0.0297 (18)	0.0510 (19)	0.0372 (19)	0.0076 (15)	0.0010 (14)	0.0010 (16)
C13B	0.0273 (17)	0.0559 (19)	0.0346 (18)	0.0078 (15)	0.0024 (14)	−0.0033 (16)
C14A	0.0284 (17)	0.0424 (17)	0.0330 (17)	0.0106 (14)	0.0013 (13)	0.0000 (14)
C14B	0.0264 (16)	0.0459 (17)	0.0283 (16)	0.0057 (14)	0.0002 (13)	0.0016 (14)
C15A	0.0331 (18)	0.0418 (17)	0.0382 (18)	0.0097 (14)	0.0028 (14)	0.0091 (14)
C15B	0.0300 (17)	0.0460 (17)	0.0345 (17)	0.0089 (14)	0.0011 (13)	0.0059 (14)
C16A	0.0382 (18)	0.0317 (15)	0.0404 (19)	0.0094 (14)	0.0037 (15)	0.0008 (14)
C16B	0.041 (2)	0.0312 (15)	0.0366 (17)	0.0085 (14)	0.0039 (15)	0.0078 (14)
C17A	0.061 (3)	0.097 (3)	0.054 (2)	−0.013 (2)	0.014 (2)	0.015 (2)
C17B	0.047 (2)	0.096 (3)	0.056 (2)	0.008 (2)	0.0145 (19)	0.029 (2)
C18A	0.068 (3)	0.0351 (17)	0.048 (2)	0.0037 (17)	−0.0059 (18)	0.0054 (16)
C18B	0.074 (3)	0.0315 (16)	0.0413 (19)	0.0017 (17)	−0.0035 (19)	0.0043 (15)
C19A	0.069 (3)	0.047 (2)	0.053 (2)	0.0155 (19)	−0.016 (2)	0.0062 (18)
C19B	0.070 (3)	0.051 (2)	0.049 (2)	0.022 (2)	−0.0089 (19)	0.0047 (17)
C20A	0.111 (4)	0.048 (2)	0.066 (3)	0.038 (3)	−0.021 (3)	−0.002 (2)
C20B	0.127 (5)	0.053 (2)	0.055 (3)	0.045 (3)	−0.012 (3)	−0.003 (2)
C21A	0.042 (2)	0.073 (3)	0.047 (2)	0.011 (2)	−0.0078 (17)	−0.015 (2)
C21B	0.044 (2)	0.071 (3)	0.047 (2)	0.006 (2)	−0.0006 (18)	−0.016 (2)

### Geometric parameters (Å, °)

**Table:**

N1A—C4A	1.487 (5)	C4B—H41B	0.9700
N1A—C5A	1.503 (4)	C4B—H42B	0.9700
N1A—H12A	0.9000	C5A—C6A	1.511 (5)
N1A—H13A	0.9000	C5A—H5A	0.9800
N1B—C5B	1.495 (4)	C5B—C6B	1.523 (4)
N1B—C4B	1.499 (5)	C5B—H5B	0.9800
N1B—H12B	0.9000	C6A—H61A	0.9700
N1B—H13B	0.9000	C6A—H62A	0.9700
N2A—C8A	1.388 (4)	C6B—H61B	0.9700
N2A—C6A	1.468 (4)	C6B—H62B	0.9700
N2A—C7A	1.479 (4)	C7A—H7A1	0.9700
N2B—C8B	1.378 (4)	C7A—H7A2	0.9700
N2B—C6B	1.467 (4)	C7B—H7B1	0.9700
N2B—C7B	1.476 (4)	C7B—H7B2	0.9700
N3A—C11A	1.346 (4)	C8A—C9A	1.403 (4)
N3A—C10A	1.404 (4)	C8A—C16A	1.413 (4)
N3A—C18A	1.470 (5)	C8B—C16B	1.406 (4)
N3B—C11B	1.347 (4)	C8B—C9B	1.410 (4)
N3B—C10B	1.406 (4)	C9A—C10A	1.410 (5)
N3B—C18B	1.469 (5)	C9B—C10B	1.408 (4)
O1A—C9A	1.377 (4)	C10A—C14A	1.409 (4)
O1A—C17A	1.428 (5)	C10B—C14B	1.399 (4)
O1B—C9B	1.375 (3)	C11A—C12A	1.361 (5)
O1B—C17B	1.436 (5)	C11A—H11A	0.9300
O2A—C13A	1.260 (4)	C11B—C12B	1.351 (5)
O2B—C13B	1.264 (4)	C11B—H11B	0.9300
O3A—C21A	1.327 (5)	C12A—C13A	1.433 (5)
O3A—H3A	0.8200	C12A—C21A	1.491 (5)
O3B—C21B	1.325 (5)	C12B—C13B	1.435 (5)
O3B—H3B	0.8200	C12B—C21B	1.484 (5)
O4A—C21A	1.201 (5)	C13A—C14A	1.447 (5)
O4B—C21B	1.211 (5)	C13B—C14B	1.450 (4)
O5A—H51A	0.8204	C14A—C15A	1.400 (4)
O5A—H52A	0.8195	C14B—C15B	1.397 (5)
O5B—H51B	0.8202	C15A—C16A	1.358 (5)
O5B—H52B	0.8204	C15A—H15A	0.9300
F1A—C16A	1.359 (3)	C15B—C16B	1.364 (5)
F1B—C16B	1.356 (3)	C15B—H15B	0.9300
C1A—C5A	1.512 (5)	C17A—H17D	0.9600
C1A—C2A	1.531 (5)	C17A—H17E	0.9600
C1A—C7A	1.534 (4)	C17A—H17F	0.9600
C1A—H1A	0.9800	C17B—H17A	0.9600
C1B—C7B	1.510 (5)	C17B—H17B	0.9600
C1B—C5B	1.523 (4)	C17B—H17C	0.9600
C1B—C2B	1.528 (5)	C18A—C19A	1.473 (6)
C1B—H1B	0.9800	C18A—C20A	1.503 (6)
C2A—C3A	1.513 (6)	C18A—H18A	0.9800
C2A—H21A	0.9700	C18B—C19B	1.483 (6)
C2A—H22A	0.9700	C18B—C20B	1.487 (6)
C2B—C3B	1.511 (5)	C18B—H18B	0.9800
C2B—H21B	0.9700	C19A—C20A	1.489 (6)
C2B—H22B	0.9700	C19A—H19C	0.9700
C3A—C4A	1.504 (5)	C19A—H19D	0.9700
C3A—H31A	0.9700	C19B—C20B	1.495 (6)
C3A—H32A	0.9700	C19B—H19A	0.9700
C3B—C4B	1.517 (5)	C19B—H19B	0.9700
C3B—H31B	0.9700	C20A—H20A	0.9700
C3B—H32B	0.9700	C20A—H20B	0.9700
C4A—H41A	0.9700	C20B—H20C	0.9700
C4A—H42A	0.9700	C20B—H20D	0.9700
			
C4A—N1A—C5A	111.6 (3)	N2B—C7B—H7B2	111.1
C4A—N1A—H12A	109.3	C1B—C7B—H7B2	111.1
C5A—N1A—H12A	109.3	H7B1—C7B—H7B2	109.1
C4A—N1A—H13A	109.3	N2A—C8A—C9A	121.1 (3)
C5A—N1A—H13A	109.3	N2A—C8A—C16A	123.1 (3)
H12A—N1A—H13A	108.0	C9A—C8A—C16A	115.8 (3)
C5B—N1B—C4B	115.2 (3)	N2B—C8B—C16B	123.9 (3)
C5B—N1B—H12B	108.5	N2B—C8B—C9B	120.4 (3)
C4B—N1B—H12B	108.5	C16B—C8B—C9B	115.7 (3)
C5B—N1B—H13B	108.5	O1A—C9A—C8A	118.4 (3)
C4B—N1B—H13B	108.5	O1A—C9A—C10A	119.7 (3)
H12B—N1B—H13B	107.5	C8A—C9A—C10A	121.7 (3)
C8A—N2A—C6A	124.6 (3)	O1B—C9B—C10B	119.3 (3)
C8A—N2A—C7A	122.3 (2)	O1B—C9B—C8B	119.2 (3)
C6A—N2A—C7A	111.1 (3)	C10B—C9B—C8B	121.4 (3)
C8B—N2B—C6B	122.2 (2)	N3A—C10A—C14A	118.3 (3)
C8B—N2B—C7B	124.3 (3)	N3A—C10A—C9A	122.4 (3)
C6B—N2B—C7B	110.9 (2)	C14A—C10A—C9A	119.3 (3)
C11A—N3A—C10A	119.5 (3)	C14B—C10B—N3B	119.0 (3)
C11A—N3A—C18A	117.3 (3)	C14B—C10B—C9B	119.5 (3)
C10A—N3A—C18A	122.6 (3)	N3B—C10B—C9B	121.5 (3)
C11B—N3B—C10B	118.8 (3)	N3A—C11A—C12A	124.4 (3)
C11B—N3B—C18B	117.5 (3)	N3A—C11A—H11A	117.8
C10B—N3B—C18B	123.0 (3)	C12A—C11A—H11A	117.8
C9A—O1A—C17A	112.1 (3)	N3B—C11B—C12B	124.6 (3)
C9B—O1B—C17B	112.3 (3)	N3B—C11B—H11B	117.7
C21A—O3A—H3A	109.5	C12B—C11B—H11B	117.7
C21B—O3B—H3B	109.5	C11A—C12A—C13A	119.7 (3)
H51A—O5A—H52A	103.2	C11A—C12A—C21A	118.5 (3)
H51B—O5B—H52B	111.0	C13A—C12A—C21A	121.6 (3)
C5A—C1A—C2A	114.4 (3)	C11B—C12B—C13B	119.9 (3)
C5A—C1A—C7A	102.2 (3)	C11B—C12B—C21B	118.6 (3)
C2A—C1A—C7A	117.2 (3)	C13B—C12B—C21B	121.4 (3)
C5A—C1A—H1A	107.5	O2A—C13A—C12A	122.6 (3)
C2A—C1A—H1A	107.5	O2A—C13A—C14A	121.8 (3)
C7A—C1A—H1A	107.5	C12A—C13A—C14A	115.7 (3)
C7B—C1B—C5B	100.3 (2)	O2B—C13B—C12B	122.6 (3)
C7B—C1B—C2B	113.2 (3)	O2B—C13B—C14B	121.7 (3)
C5B—C1B—C2B	112.2 (3)	C12B—C13B—C14B	115.7 (3)
C7B—C1B—H1B	110.3	C15A—C14A—C10A	118.7 (3)
C5B—C1B—H1B	110.3	C15A—C14A—C13A	119.6 (3)
C2B—C1B—H1B	110.3	C10A—C14A—C13A	121.7 (3)
C3A—C2A—C1A	113.3 (3)	C15B—C14B—C10B	118.9 (3)
C3A—C2A—H21A	108.9	C15B—C14B—C13B	119.9 (3)
C1A—C2A—H21A	108.9	C10B—C14B—C13B	121.1 (3)
C3A—C2A—H22A	108.9	C16A—C15A—C14A	120.3 (3)
C1A—C2A—H22A	108.9	C16A—C15A—H15A	119.8
H21A—C2A—H22A	107.7	C14A—C15A—H15A	119.8
C3B—C2B—C1B	111.5 (3)	C16B—C15B—C14B	120.2 (3)
C3B—C2B—H21B	109.3	C16B—C15B—H15B	119.9
C1B—C2B—H21B	109.3	C14B—C15B—H15B	119.9
C3B—C2B—H22B	109.3	C15A—C16A—F1A	117.2 (3)
C1B—C2B—H22B	109.3	C15A—C16A—C8A	123.4 (3)
H21B—C2B—H22B	108.0	F1A—C16A—C8A	119.4 (3)
C4A—C3A—C2A	111.7 (3)	F1B—C16B—C15B	117.1 (3)
C4A—C3A—H31A	109.3	F1B—C16B—C8B	119.5 (3)
C2A—C3A—H31A	109.3	C15B—C16B—C8B	123.3 (3)
C4A—C3A—H32A	109.3	O1A—C17A—H17D	109.5
C2A—C3A—H32A	109.3	O1A—C17A—H17E	109.5
H31A—C3A—H32A	107.9	H17D—C17A—H17E	109.5
C2B—C3B—C4B	111.0 (3)	O1A—C17A—H17F	109.5
C2B—C3B—H31B	109.4	H17D—C17A—H17F	109.5
C4B—C3B—H31B	109.4	H17E—C17A—H17F	109.5
C2B—C3B—H32B	109.4	O1B—C17B—H17A	109.5
C4B—C3B—H32B	109.4	O1B—C17B—H17B	109.5
H31B—C3B—H32B	108.0	H17A—C17B—H17B	109.5
N1A—C4A—C3A	109.7 (3)	O1B—C17B—H17C	109.5
N1A—C4A—H41A	109.7	H17A—C17B—H17C	109.5
C3A—C4A—H41A	109.7	H17B—C17B—H17C	109.5
N1A—C4A—H42A	109.7	N3A—C18A—C19A	118.4 (3)
C3A—C4A—H42A	109.7	N3A—C18A—C20A	115.7 (3)
H41A—C4A—H42A	108.2	C19A—C18A—C20A	60.0 (3)
N1B—C4B—C3B	109.8 (3)	N3A—C18A—H18A	116.9
N1B—C4B—H41B	109.7	C19A—C18A—H18A	116.9
C3B—C4B—H41B	109.7	C20A—C18A—H18A	116.9
N1B—C4B—H42B	109.7	N3B—C18B—C19B	118.0 (3)
C3B—C4B—H42B	109.7	N3B—C18B—C20B	116.7 (3)
H41B—C4B—H42B	108.2	C19B—C18B—C20B	60.4 (3)
N1A—C5A—C6A	112.8 (3)	N3B—C18B—H18B	116.6
N1A—C5A—C1A	110.6 (3)	C19B—C18B—H18B	116.6
C6A—C5A—C1A	103.9 (3)	C20B—C18B—H18B	116.6
N1A—C5A—H5A	109.8	C18A—C19A—C20A	61.0 (3)
C6A—C5A—H5A	109.8	C18A—C19A—H19C	117.7
C1A—C5A—H5A	109.8	C20A—C19A—H19C	117.7
N1B—C5B—C6B	114.2 (2)	C18A—C19A—H19D	117.7
N1B—C5B—C1B	112.9 (2)	C20A—C19A—H19D	117.7
C6B—C5B—C1B	104.4 (3)	H19C—C19A—H19D	114.8
N1B—C5B—H5B	108.4	C18B—C19B—C20B	59.9 (3)
C6B—C5B—H5B	108.4	C18B—C19B—H19A	117.8
C1B—C5B—H5B	108.4	C20B—C19B—H19A	117.8
N2A—C6A—C5A	103.5 (3)	C18B—C19B—H19B	117.8
N2A—C6A—H61A	111.1	C20B—C19B—H19B	117.8
C5A—C6A—H61A	111.1	H19A—C19B—H19B	114.9
N2A—C6A—H62A	111.1	C19A—C20A—C18A	58.9 (3)
C5A—C6A—H62A	111.1	C19A—C20A—H20A	117.9
H61A—C6A—H62A	109.0	C18A—C20A—H20A	117.9
N2B—C6B—C5B	102.5 (2)	C19A—C20A—H20B	117.9
N2B—C6B—H61B	111.3	C18A—C20A—H20B	117.9
C5B—C6B—H61B	111.3	H20A—C20A—H20B	115.0
N2B—C6B—H62B	111.3	C18B—C20B—C19B	59.6 (3)
C5B—C6B—H62B	111.3	C18B—C20B—H20C	117.8
H61B—C6B—H62B	109.2	C19B—C20B—H20C	117.8
N2A—C7A—C1A	103.1 (2)	C18B—C20B—H20D	117.8
N2A—C7A—H7A1	111.1	C19B—C20B—H20D	117.8
C1A—C7A—H7A1	111.1	H20C—C20B—H20D	114.9
N2A—C7A—H7A2	111.1	O4A—C21A—O3A	121.4 (4)
C1A—C7A—H7A2	111.2	O4A—C21A—C12A	124.4 (4)
H7A1—C7A—H7A2	109.1	O3A—C21A—C12A	114.1 (4)
N2B—C7B—C1B	103.4 (2)	O4B—C21B—O3B	121.8 (4)
N2B—C7B—H7B1	111.1	O4B—C21B—C12B	123.6 (4)
C1B—C7B—H7B1	111.1	O3B—C21B—C12B	114.5 (4)
			
C5A—C1A—C2A—C3A	43.5 (4)	C8B—C9B—C10B—C14B	10.7 (5)
C7A—C1A—C2A—C3A	−76.1 (4)	O1B—C9B—C10B—N3B	15.1 (5)
C7B—C1B—C2B—C3B	−164.7 (3)	C8B—C9B—C10B—N3B	−169.0 (3)
C5B—C1B—C2B—C3B	−52.1 (4)	C10A—N3A—C11A—C12A	4.8 (5)
C1A—C2A—C3A—C4A	−48.2 (4)	C18A—N3A—C11A—C12A	−166.2 (4)
C1B—C2B—C3B—C4B	57.9 (4)	C10B—N3B—C11B—C12B	−5.8 (5)
C5A—N1A—C4A—C3A	−62.9 (4)	C18B—N3B—C11B—C12B	165.0 (4)
C2A—C3A—C4A—N1A	57.7 (5)	N3A—C11A—C12A—C13A	3.1 (6)
C5B—N1B—C4B—C3B	53.3 (3)	N3A—C11A—C12A—C21A	178.2 (3)
C2B—C3B—C4B—N1B	−57.2 (4)	N3B—C11B—C12B—C13B	−2.4 (5)
C4A—N1A—C5A—C6A	173.1 (3)	N3B—C11B—C12B—C21B	−178.0 (4)
C4A—N1A—C5A—C1A	57.2 (3)	C11A—C12A—C13A—O2A	173.9 (3)
C2A—C1A—C5A—N1A	−47.1 (4)	C21A—C12A—C13A—O2A	−1.1 (5)
C7A—C1A—C5A—N1A	80.7 (3)	C11A—C12A—C13A—C14A	−6.4 (5)
C2A—C1A—C5A—C6A	−168.4 (3)	C21A—C12A—C13A—C14A	178.7 (3)
C7A—C1A—C5A—C6A	−40.7 (3)	C11B—C12B—C13B—O2B	−174.0 (3)
C4B—N1B—C5B—C6B	70.6 (4)	C21B—C12B—C13B—O2B	1.5 (5)
C4B—N1B—C5B—C1B	−48.5 (4)	C11B—C12B—C13B—C14B	5.5 (5)
C7B—C1B—C5B—N1B	167.1 (3)	C21B—C12B—C13B—C14B	−179.0 (3)
C2B—C1B—C5B—N1B	46.7 (4)	N3A—C10A—C14A—C15A	−176.0 (3)
C7B—C1B—C5B—C6B	42.5 (3)	C9A—C10A—C14A—C15A	5.7 (4)
C2B—C1B—C5B—C6B	−78.0 (3)	N3A—C10A—C14A—C13A	5.3 (5)
C8A—N2A—C6A—C5A	−175.1 (3)	C9A—C10A—C14A—C13A	−173.1 (3)
C7A—N2A—C6A—C5A	−11.3 (4)	O2A—C13A—C14A—C15A	3.2 (5)
N1A—C5A—C6A—N2A	−87.7 (3)	C12A—C13A—C14A—C15A	−176.5 (3)
C1A—C5A—C6A—N2A	32.2 (4)	O2A—C13A—C14A—C10A	−178.0 (3)
C8B—N2B—C6B—C5B	−157.2 (3)	C12A—C13A—C14A—C10A	2.2 (5)
C7B—N2B—C6B—C5B	5.5 (3)	N3B—C10B—C14B—C15B	175.7 (3)
N1B—C5B—C6B—N2B	−153.7 (3)	C9B—C10B—C14B—C15B	−4.0 (5)
C1B—C5B—C6B—N2B	−29.9 (3)	N3B—C10B—C14B—C13B	−7.0 (4)
C8A—N2A—C7A—C1A	150.5 (3)	C9B—C10B—C14B—C13B	173.3 (3)
C6A—N2A—C7A—C1A	−13.7 (4)	O2B—C13B—C14B—C15B	−4.1 (5)
C5A—C1A—C7A—N2A	33.0 (3)	C12B—C13B—C14B—C15B	176.4 (3)
C2A—C1A—C7A—N2A	158.9 (3)	O2B—C13B—C14B—C10B	178.7 (3)
C8B—N2B—C7B—C1B	−176.7 (3)	C12B—C13B—C14B—C10B	−0.8 (4)
C6B—N2B—C7B—C1B	21.1 (4)	C10A—C14A—C15A—C16A	1.7 (5)
C5B—C1B—C7B—N2B	−38.1 (3)	C13A—C14A—C15A—C16A	−179.5 (3)
C2B—C1B—C7B—N2B	81.6 (3)	C10B—C14B—C15B—C16B	−3.7 (5)
C6A—N2A—C8A—C9A	−162.1 (3)	C13B—C14B—C15B—C16B	178.9 (3)
C7A—N2A—C8A—C9A	35.9 (5)	C14A—C15A—C16A—F1A	176.0 (3)
C6A—N2A—C8A—C16A	18.8 (5)	C14A—C15A—C16A—C8A	−5.6 (5)
C7A—N2A—C8A—C16A	−143.3 (3)	N2A—C8A—C16A—C15A	−179.1 (3)
C6B—N2B—C8B—C16B	150.5 (3)	C9A—C8A—C16A—C15A	1.7 (5)
C7B—N2B—C8B—C16B	−9.8 (5)	N2A—C8A—C16A—F1A	−0.7 (5)
C6B—N2B—C8B—C9B	−31.3 (5)	C9A—C8A—C16A—F1A	−180.0 (3)
C7B—N2B—C8B—C9B	168.4 (3)	C14B—C15B—C16B—F1B	−176.7 (3)
C17A—O1A—C9A—C8A	94.2 (4)	C14B—C15B—C16B—C8B	5.2 (5)
C17A—O1A—C9A—C10A	−80.8 (4)	N2B—C8B—C16B—F1B	1.4 (5)
N2A—C8A—C9A—O1A	11.8 (5)	C9B—C8B—C16B—F1B	−176.9 (3)
C16A—C8A—C9A—O1A	−168.9 (3)	N2B—C8B—C16B—C15B	179.4 (3)
N2A—C8A—C9A—C10A	−173.2 (3)	C9B—C8B—C16B—C15B	1.2 (5)
C16A—C8A—C9A—C10A	6.0 (5)	C11A—N3A—C18A—C19A	118.3 (4)
C17B—O1B—C9B—C10B	91.9 (4)	C10A—N3A—C18A—C19A	−52.5 (5)
C17B—O1B—C9B—C8B	−84.1 (4)	C11A—N3A—C18A—C20A	50.0 (5)
N2B—C8B—C9B—O1B	−11.5 (5)	C10A—N3A—C18A—C20A	−120.7 (4)
C16B—C8B—C9B—O1B	166.8 (3)	C11B—N3B—C18B—C19B	−117.0 (4)
N2B—C8B—C9B—C10B	172.6 (3)	C10B—N3B—C18B—C19B	53.3 (5)
C16B—C8B—C9B—C10B	−9.1 (5)	C11B—N3B—C18B—C20B	−48.0 (5)
C11A—N3A—C10A—C14A	−8.8 (5)	C10B—N3B—C18B—C20B	122.3 (4)
C18A—N3A—C10A—C14A	161.8 (3)	N3A—C18A—C19A—C20A	−104.9 (4)
C11A—N3A—C10A—C9A	169.5 (3)	N3B—C18B—C19B—C20B	106.4 (4)
C18A—N3A—C10A—C9A	−19.9 (5)	N3A—C18A—C20A—C19A	109.4 (4)
O1A—C9A—C10A—N3A	−13.1 (5)	N3B—C18B—C20B—C19B	−108.6 (4)
C8A—C9A—C10A—N3A	172.0 (3)	C11A—C12A—C21A—O4A	3.9 (6)
O1A—C9A—C10A—C14A	165.2 (3)	C13A—C12A—C21A—O4A	179.0 (4)
C8A—C9A—C10A—C14A	−9.7 (5)	C11A—C12A—C21A—O3A	−174.5 (3)
C11B—N3B—C10B—C14B	10.3 (5)	C13A—C12A—C21A—O3A	0.5 (5)
C18B—N3B—C10B—C14B	−159.9 (3)	C11B—C12B—C21B—O4B	−4.8 (6)
C11B—N3B—C10B—C9B	−170.0 (3)	C13B—C12B—C21B—O4B	179.7 (4)
C18B—N3B—C10B—C9B	19.8 (5)	C11B—C12B—C21B—O3B	174.5 (3)
O1B—C9B—C10B—C14B	−165.2 (3)	C13B—C12B—C21B—O3B	−1.0 (5)

### Hydrogen-bond geometry (Å, °)

**Table:**

*D*—H···*A*	*D*—H	H···*A*	*D*···*A*	*D*—H···*A*
N1A—H12A···Cl1B^i^	0.90	2.26	3.113 (3)	158
N1A—H13A···Cl1A	0.90	2.43	3.234 (3)	150
N1B—H12B···Cl1A^ii^	0.90	2.22	3.109 (3)	170
N1B—H13B···Cl1B	0.90	2.25	3.114 (2)	162
O3A—H3A···O2A	0.82	1.74	2.512 (4)	156
O3B—H3B···O2B	0.82	1.74	2.508 (4)	155
O5A—H51A···O4A	0.82	2.24	3.011 (5)	157
O5A—H52A···Cl1B	0.82	2.72	3.422 (5)	144
O5B—H51B···O4B	0.82	2.12	2.911 (5)	161
O5B—H52B···Cl1B^iii^	0.82	2.41	3.208 (4)	163
C1A—H1A···O3B	0.98	2.53	3.373 (4)	144
C2A—H21A···Cl1A^iv^	0.97	2.80	3.742 (4)	163
C3A—H31A···Cl1A	0.97	2.74	3.518 (5)	138
C3A—H32A···O1B^v^	0.97	2.59	3.450 (5)	148
C6A—H61A···O3B^vi^	0.97	2.49	3.340 (6)	146
C18B—H18B···O5A^vii^	0.98	2.58	3.564 (7)	179

Symmetry codes: (i) *x*, *y*+1, *z*+1; (ii) *x*, *y*, *z*−1; (iii) *x*+1, *y*+1, *z*+1; (iv) *x*+1, *y*, *z*; (v) *x*, *y*, *z*+1; (vi) *x*−1, *y*, *z*; (vii) *x*+1, *y*+1, *z*.
